# Prevalence of soil transmitted helminths in school-aged children, Colombia, 2012-2013

**DOI:** 10.1371/journal.pntd.0007613

**Published:** 2020-07-17

**Authors:** David José González Quiroz, Sonia del Pilar Agudelo Lopez, Catalina María Arango, Jesús Ernesto Ochoa Acosta, León Darío Bello Parias, Leonardo Uribe Alzate, Carolina Hernández Castro, Angélica Patricia Medina Lozano, Geicy Derly Sepúlveda Vergara, Adriana Molina Giraldo, Julián Trujillo-Trujillo, Ivet Del Carmen Pernett Bolaño, Claudia Milena Cuellar Segura, María Patricia Arbeláez Montoya

**Affiliations:** 1 Epidemiology Group, National School of Public Health, University of Antioquia, Medellin, Colombia; 2 Parasitology Group, School of Medicine, University of Antioquia, Medellin, Colombia; 3 Promotion and Prevention Department, Ministry of Health and Social Protection, Bogotá, Colombia; 4 Knowledge Management Group, Epidemiology and Demography Department, Ministry of Health and Social Protection, Bogotá, Colombia; Texas A&M University College Station, UNITED STATES

## Abstract

**Background:**

This study aims to establish the prevalence of soil-transmitted helminth (STH) intestinal infections, nutritional status, and anemia in school children aged 7 to 10 years old in the biogeographic provinces of Colombia in 2012–2013. STH prevalence in the country has not been described within the last 30 years and it is needed in order to establish policies its control in the country.

**Methodology:**

National Survey of STH in school-aged children with a multistage stratified probability sampling was conducted. The overall prevalence and intensity of STH infection, as well as for each parasite, (*A*. *lumbricoides*, *T*. *trichiura* and hookworms) were calculated for the country and for each of the nine biogeographic provinces.

**Principal findings:**

Stool samples were collected from 6045 children in eight out of nine biogeographic provinces. The combined prevalence of STH in the country was 29.6%. *T*. *trichiura* was the most prevalent helminth (18.4%), followed by *A*. *lumbricoides* (11.3%), and hookworms (6.4%). For *A*. *lumbricoides* and hookworms, the highest prevalence values were found in the *Amazonía* province (58.0% and 35.7%, respectively). Regarding STH intensity, most cases showed moderate intensity (41.3%) for *A*. *lumbricoides*, and light intensity, for *T*. *trichiura* and hookworms. The national prevalence of anemia in school-aged children was 14.2%, lowest in the *Nor-Andina* province (3.5%), and highest in the *Territorios Insulares oceánicos del Caribe* province (45.1%).

**Significance:**

Colombia has a moderate risk of STH infection in school-aged populations, with considerable variation in the prevalence values among the biogeographic provinces. Like any public health issue, this problem should be handled with a comprehensive approach that involves deworming programs and strategies for STH control according to the specific epidemiological and socioeconomic conditions and sanitation service coverage in each biogeographic province. The program should be further supported by intersectoral action to improve living conditions, particularly the excreta disposal, promoted at municipality levels.

## Author summary

STH infection (infection by *A*. *lumbricoides*, *T*. *trichiura*, and hookworms) are endemic in Colombia; however, its prevalence in the country has not been described within the last 30 years. This study determines the prevalence and intensity of STH infections, nutritional status, and anemia in children, using multistage stratified probability sampling. The prevalence result of all STH at national level was 29.6%. *T*. *trichiura* was the most prevalent (18.4%), followed by *A*. *lumbricoides* (11.3%), and hookworms (6.4%). Comparing the biogeographic provinces (eco-epidemiological zones), *Amazonía* had the highest STH prevalence. A large spatial variation was found in STH prevalence by province. The study shows that STH infection remains a national public health problem, which requires intervention through nationwide strategies using a comprehensive inter-programmatic approach and prioritizing high-risk areas. These strategies should aim at improving the living conditions associated with the infection while being supported by large-scale anthelmintic chemotherapy.

## Introduction

Soil-transmitted helminth (STH) intestinal infections are among the diseases with the highest prevalence and morbidity in vulnerable populations of developing countries. Although these infections can affect people of all ages, high-risk groups are pre-school and school-aged children as well as women of child bearing age including pregnant women after the first trimester [1,2]. The estimates have shown that in 2010, 819 million people were infected with *A*. *lumbricoides*, 465 million with *T*. *trichiura*, and 439 million with hookworms worldwide. The greatest numbers of STH infections occur in the Americas, China and East Asia, and Sub-Saharan Africa. [3]

The morbidity caused by STH is most commonly associated with infections of heavy intensity. Of 3.3 million estimated years lived with disability due to STH globally in 2010, Latin American countries represented 11.3%. There were 2,824 deaths attributable to *A*. *lumbricoides* in 2010. [3]

Chronic STH infections are associated with growth retardation, weight loss, anemia, school absenteeism, and poor academic performance. In addition to their effect on physical health, helminth infections can also impair mental growth in childhood and hinder their economic development. [4,5]

Literature has not been reviewed for recent evidence of the general situation of intestinal parasitism in Colombia. Related data have not been reviewed nor reported since 1980. Occasional studies in different parts of the country have found that the combined STH prevalence ranges from 10.7% to 49.3% [5,6]. Therefore, an investigation into the current prevalence of STH in the country is essential to create the baseline data that are required to formulate effective STH surveillance and implement control programs proposed by the World Health Organization (WHO). The aim of this study is to determine the prevalence of STH infection and its intensity in Colombia in addition to the nutritional status and patterns of anemia, citing the differences among the biogeographic provinces of the country. This, in turn, would support decision makers in developing focalized programmatic interventions to effectively decrease the STH morbidity in the country.

## Materials and methods

### Ethics statement

The study protocol had the approval of the Institutional Review Board at the School of Public Health, University of Antioquia. It also complied with the provisions in the Resolution 008430 1993 from the Colombian Ministry of Health. The children and their parents or caregivers were informed about the purpose of this study, the procedures to be carried out, and the implications of the results. After a group meeting, explanation at each class including, professor, parents, caregivers and research team members, forms of survey questionnaire, informed consent for parents, and assent for children were provided and read to them by a research team member and then signed. Also, we gave them copies of the consent forms. We keep originals with the signatures. The children who were found to be carrying other intestinal parasites that required medical treatment were referred to their healthcare providers, with the corresponding recommendation in the result report that we sent to each school.

### Type of study

This investigation was a cross-sectional study using a population survey with multistage stratified random sampling. It combined three sampling stages, where each unit of analysis had a known probability of being selected.

### Population

The population used for calculating the sample size was 3,056,628 children of age 7 to 10 estimated to be enrolled in school between 2012 and 2013. The estimation was based on population projections by the National Department of Statistics (DANE) for 2012, assuming 89% of these children would be enrolled in school [7].

The WHO recommendations were followed, whenever possible, in this study focused on third-grade children in Colombia. This age group (7 to 10 years old) is likely to have a high burden of STH infection, and thus can be considered a reflection on local conditions. Their inclusion is more feasible than others because of ease in school recruitment [6].

Colombia is divided into nine biogeographic provinces: *I)*. *Territorios Insulares Oceánicos del Caribe*: This province has a warm and humid climate, which is dominated by dry vegetation and a mix of Central American and West Indian elements. *II)*. *Territorios Insulares Oceánicos del Pacífico*: It lacks permanent human settlements; therefore, it was not included in this study. *III)*. *Cinturón Árido Pericaribeño*: It is characterized by a warm climate as well as a system of seasonal rains and prolonged dry spells. *IV)*. *Sierra Nevada de Santa Marta*: In addition to the humid weather, this province has the widest range of altitudes and temperatures in Colombia since it is a highly mountain region bordering the Caribbean Sea. *V)*. *Chocó-Magdalena*: Its climate is warm and humid and its vegetation largely a tropical rainforest. *VI)*. *La Orinoquía*: It has a predominantly hot and dry climate with the northern region receiving more moisture. *VII)*. *La Guayana*: This province´s climate is predominantly warm and humid with moisture increasing in the southern direction. *VIII)*. *La Amazonía*: This is the province of tropical rainforests with a predominantly hot and humid to very humid climate. *IX)*. *Nor-Andina*: It is comprised of the three mountain ranges and valleys in los Andes in Colombia and the highest average height above sea level (2160 meters) [8]. It has the highest population density in the country. Large cities with more than 100,000 inhabitants, including the capitals were excluded from sampling in this study since they require a more complex sampling process. However, we included small municipalities because they are located mostly in rural areas where unsanitary conditions are more frequent. (S1 Fig).

### Sample design

A pilot study was conducted in four states of the country in order to adjust and standardize the methodology and instruments used for data collection. The proportion of STH infections by *A*. *lumbricoides* prevalence (p) found in the pilot study were employed to calculate the sample size used in this study. Calculations of the sample size were done for each biogeographic province using the frequency of *A*. *lumbricoides* found in a previous pilot study (p = 16% for province IX; p = 17% for provinces I, III, IV, V; and p = 74% for the remaining provinces).

After a sample calculation of n = 6,128 children of ages 7–10 in the country (including the number calculated for each province), a multistage stratified sampling was used in three stages. First stage: Within biogeographic provinces I and III to IX (strata), we conducted a random selection of municipalities proportional to their size from the total municipalities in each biogeographic province. Second stage: From the list of schools provided by the education secretariat at each municipality, (cluster) we randomly selected at least one urban school and one rural school, or additional ones if required, to cover the sample size estimate of each municipality. Third stage: For the random selection of school classes (sampling unit) we selected the third-grade class since it is composed of children aged 7 to 10 years old on average. We included all children from each class selected. (S1 Table)

### Inclusion criteria

The cohort of this study consisted of school children aged 7 to 10 years old, accompanied by their parents or caregivers and their identification cards. Permission, both from the parent or caregiver and the child, was expressed by signing a consent and assent form. We interviewed them with a questionnaire that included demographics, sanitary conditions, clinical manifestations, and hygiene practices, among other variables. The analysis only included the children that had signed consent and assent forms, completed surveys, and provided stool samples.

### Field work

We formed three field work groups, each comprising 6 persons: a physician, a nurse, a bacteriologist, and three assistants. All the members in each group were previously standardized in the study data collection and reinforced the training before each field visit. The field work was performed in parallel by the three groups in November 2012, February 2013, and between September to November 2013. These phases were performed due to the disbursement of financial resources in different periods of time, schoolchildren´s vacation periods and social conflicts in some rural regions.

### Weight, height, and hemoglobin measurement

Measurement of body weight was carried out using 12 digital portable scale (TANITA model HD313) with a sensitivity of 100 g and a capacity of 150 kg. Height measurement was performed by six portable stadiometers (SECA) with a maximum length of 2.20 m and a sensitivity of 1 mm. Three devices of HemoCue® portable photometer Hb 201+ with a measuring range from 0 to 25.6 g/dl, were used to measure hemoglobin; the presence of anemia was indicated by Hb < 11.5 g/dl, according to WHO recommendation criteria [9]. Each device was properly calibrated (Eurotrol Hemotrol reference 171.003.002) and the staff adopted a standardization policy by using a previous elaboration of a technical manual.

Anthropometry measurements were taken and classified following the WHO standards, including data of Body Mass Index. BMI/Age was calculated as the square of the ratio between weight in kilograms and height in centimeters for the child´s age [10]. Children were then classified, into the following nutritional status categories: Overweight, > = + 1 SD; Obesity, > = + 2 SD; Thinness, < = - 2 SD; Severe thinness, < = - 3 SD [11].

### Laboratory tests

For the parasitological study, we collected only one stool specimen obtained by fecal excretion from each child. Specimens of fresh feces (without preservatives) were packed according to IATA (International Air Transport Association) standards labeled stool specimens and the corresponding tracking sheets were sent by air plane to the Laboratory of Parasitology, School of Medicine, University of Antioquia during the day of collection (before 24 hours) using ice packs.

In the lab, the mounting and reading of the specimens were done in two phases. In the first phase, techniques that require a fresh stool specimen, such as Kato-Katz were mounted within the first 24 hours and immediately read. Next, an agar isolation was mounted within 48 hours. After performing the Kato-Katz reading, 5% formalin was added to each specimen for preservation. In the second phase, direct stool, formalin-ether concentration, and agar isolation were read and processed. Thus, every stool specimen was completely processed and read within an estimated time of 30 days.

The National Parasitism Survey conducted in Colombia 2012–2013 included different species of helminths, protozoa and chromist. For this paper we included only helminths and identified them through four lab techniques: Kato-Katz (nigrosine-eosin) modified to keep Hookworms egg readings possible several days after, direct stool, formalin-ether concentration (Ritchie modification), and agar isolation technique for *Strongyloides stercoralis* identification.[6,12]

In order to improve the diagnostic sensitivity, the prevalence was estimated based on a minimum of one positive result obtained by any of these techniques, reducing potential information underestimation [13].

Helminth intensity (eggs per gram) was measured according WHO criteria, for *Ascaris lumbricoides*: light 1–4,999, Moderated 5,000–49,999 and heavy > 50,000. *Trichuris trichiura*: light 1–999, Moderated 1,000–9,999 and heavy > 10,000. Hookworms: light 1–1,999, Moderated 2,000–3,999 and heavy > 4,000.[6]

### Deworming

Every participating child in the survey was given an antiparasitic treatment comprised of one dose of albendazole, 400 mg. However, children who had received treatment within the previous three months were not given the treatment immediately; these children received the albendazole three or more months after the previous treatment, following recommendations given by us to their parents [2].

### Quality control

An experienced bacteriologist designated by the Laboratory of Parasitology, School of Medicine, University of Antioquia, conducted an internal quality control on 3% of the processed samples using the concentration technique. Additionally, the Colombian National Health Institute conducted an external quality control on 10% of the negative samples and all the positive ones by analyzing one half of the sediment that was previously used in the concentration technique. The external quality had a high agreement index of more than 80% in all cases as well as a Cohen´s Kappa coefficient of 0.91 (95%CI: 0.89–0.92) for *A*. *lumbricoides*, 0.79 (95%CI: 0.77–0.81) for *T*. *trichiura*, and 0.79 (95%CI: 0.76–0.83) for hookworms. For prevalence estimates, we referenced the positive results from the laboratory in the University of Antioquia.

### Return of results

The participating schools received the results of stool analysis for each child, the data for the measurements of weight, height, and hemoglobin along with the corresponding nutritional status, as well as nutritional and health recommendations. A summary overview of the results was also sent to the corresponding municipal health authority and education secretariats. Those mechanisms were included in the informed consent form.

### Statistical procedures

Data analysis was performed using a package for the Social Sciences 22 (SPSS Inc., Chicago, Illinois) and ArcGIS 10 software (ESRI, Redlands, CA, USA). The prevalence and intensity of infection was calculated for each STH (*A*. *lumbricoides*, *T*. *trichiura*, and hookworms) for the country and for each biogeographic province, with a respective 95% confidence interval (CI), using the complex sample module in SPSS. Additionally, the combined STH prevalence (P *a*.*t*.*h*.) was calculated for each province using the formula recommended by WHO [6,14]:

$$P\;a.t.h.=\frac{\left(a+t+h)-(a*t+a*h+t*h)+(a*t*h\right)}{1.06}*100$$

Where:

*a* = *A*. *lumbricoides* prevalence (expressed as a proportion),

*t* = *T*. *trichiura* prevalence (expressed as a proportion),

*h* = hookworm’s prevalence (expressed as a proportion),

Expansion factors were calculated to adjust the prevalence ratio of each STH, the proportions of anemia, and the nutritional status categories by biogeographic province.

## Results

### Overview of the study population

Stool specimens were collected from 6,045 of 7,860 surveyed children (76.9%). The distribution in each biogeographic province according to the sampling design is shown in S1 Table.

The study population had a homogeneous distribution in terms of the gender of participants; 2,951 (48.8%) boys and 3,094 (51.2%) girls. 2,529 (38.6%) of the population were found to live in rural areas. The entire population in province I (*Territorios Insulares Oceánicos del Caribe*) was found to be living in rural areas. 4,762 (74.5%) of the children were taken care of by their parents. 1,255 (24.4%) of parents had completed high school, and only 273 (6.8%) had college degrees.

5,694 (94.8%) of all persons were affiliated with the General System of Social Security in Health (GSSSH), and 4,531 (61.4%) had subsidized health coverage. Furthermore, 5,214 (72.4%) of the population belonged to the two lowest socioeconomic strata (1 and 2), in Colombia we define 6 socioeconomic strata, strata 1 and 2 being the lowest.

4,677 (64.5%) of the households were found to earn below the monthly minimum wage (MMW). Provinces III, *Cinturón Arido Pericaribeño*, IV, *Sierra Nevada de Santa Marta*, and VII, *La Guayana*, had the lowest household monthly income. 3,135 (44.3%) of the population reported that their monthly income falls short of covering their minimum household expenses.

3,939 (57.7%) of the population expressed concern about their low income and capability to provide food. Meanwhile, 2,560 (32.6%) reported that the lack of money resulted in a reduction of the quality of nutritious meals or meal portions. Nevertheless, most of them, precisely 4,968 (87.2%) of participants, reported that the situation never meant that the children had to skip any main meals.

### Clinical aspects

Regarding weight and height, 4,715 (78.4%) of the children were found in the normal nutritional status, and 19.5% were found to be in the overweight or obesity range. The national prevalence of anemia was 14.2% (n = 1,176); IX (*Nor-Andina*: 3.5%, n = 143) being the lowest of the provinces and the highest being in province I (*Territorios Insulares Oceánicos del Caribe*: 45.1%, n = 14) (S2 Table).

22.1% of children responded that they had received deworming treatment during the last three months before the data collection; the STH combined prevalence was 21,3% (935/4381) in children with self-reported purgative and 27,6% (459/1664), in children who did not report deworming during that period. The highest proportions were found in provinces IV (*Sierra Nevada de Santa Marta*: 44.4%) and I (*Territorios Insulares Oceánicos del Caribe*: 36.3%). 27% of those children who had received deworming medication used a home purgative or were self-medicated.

### Prevalence and intensity of STH infection

Our final sample size was n = 6,045, lower than the calculated 6,128, due to difficulties in the rural provinces (I, IV, VI, VII, and VIII) which have fewer municipalities, dispersed population, and smaller school classrooms, contrary to the other provinces (III, V, and IX) where we included more children than expected due to bigger school classrooms.

We performed the Kato-Katz technique on 5,926 stool samples, direct stool on 6,045, stool by formalin-ether concentration on 6,045, and agar isolation technique on 5,795.

The combined prevalence of STH in the country was 29.6%. *T*. *trichiura* was the most prevalent helminth (18.4%, 95%CI: 11.8–27.7), followed by *A*. *lumbricoides* (11.3%, 95%CI: 7.1–17.4), and hookworms (6.4%, 95%CI: 3.7–10.8). Comparing the biogeographic provinces, the highest prevalence of *T*. *trichiura* was detected in province IV (*Sierra Nevada de Santa Marta*: 61.0%), followed by province VIII (*La Amazonía*: 50%), and lastly in province III (*Cinturón Arido Pericaribeño*: 42.7%), (S1 Table, S2 Fig). The highest prevalence values for *A*. *lumbricoides* and hookworms were found in province VIII (*La Amazonía*: 58.0% and 35.7%, respectively), (S3 Fig).

The parasitic intensity in the country was generally light for *T*. *trichiura* (59.0%) and hookworms (86.4%). For *A*. *lumbricoides*, most cases were of moderate intensity (41.3%). The heaviest intensity of *A*. *lumbricoides* in the country was found in province VIII (*La Amazonía*: 35% of school-aged children).

On the country level, *S*. *stercoralis* had a significantly lower prevalence (0.7%) than the three others main STHs, and its highest prevalence (4.4%) was found in province VIII, *La Amazonia*. Moreover, *E*. *vermicularis* had a prevalence of 1.0% in the country and had its highest prevalence in province VII, *La Guayana* (6.4%). The prevalence of *Hymenolepsis nana* was less than 1.0% in the country and only five cases of *Taenia solium/saginata* were found. It is relevant to note that this study did not use reference diagnostic techniques for these helminths.

## Discussion

The National Survey of STH infections in elementary school-aged children, Colombia 2012–2013, is the third population-based study conducted in the country to assess the status of these infections. It was designed and performed with methodological characteristics, which are different from those used in the National Morbidity Survey 1966 (NMS 1966) and the National Health Survey 1980 (NHS 1980). Hence, the data of this study are difficult to compare with those acquired in the previous surveys [15]. Besides, our study excluded cities with more than 100,000 inhabitants reducing our inference to urban capital cities.

The obtained prevalence confirms that all the agents that cause STH infections are endemic in Colombia. Many explanations can account for these results one being the country’s location in the tropics, with mean temperatures of 75.2°F and the warm climatic zones in 82% of the Colombian territory located less than a 1,000 m above sea level. Moreover, the social conditions in some areas of the country can facilitate the life cycle of STHs, allowing their active transmission and persistence in the environment. Lastly, the country lacks a robust prevention and control program, which would entail a need for financial, logistical, and human resources in order to sustain an effective intervention required for STH control [16]. Children who self-reported deworming during the last three months had a significant reduction in STH combined prevalence. This highlight the importance of implementing mass antiparasitic campaigns, as public health activities in the country.

Despite the methodological differences among the three national surveys, the findings frequently infer that *T*. *trichiura* is the most prevalent intestinal worm in Colombia, followed by *A*. *lumbricoides* and hookworms. However, a decrease in the prevalence of these STH can be observed when comparing the prevalence obtained here to those found in the previous surveys for the same age range. Colombia was previously classified as a high-risk area, while the current results allow one to classify the country as an area of moderate risk of infection (NMS 1966: *A*. *lumbricoides* 65.1%, *T*. *trichiura* 61.6%, and hookworms 25%; NHS 1980: *A*. *lumbricoides* 45.1%, *T*. *trichiura* 51.6%, and hookworms 24.8%) [17].

This decrease in the prevalence of STH infections is most likely associated to an improvement in living conditions, mostly in the *Nor-Andina* biographical province, and to the frequent use of deworming treatments, even without medical prescription. Nevertheless, regional differences persist, and the frequency of reinfection and its relationship with sanitation in the Colombian municipalities has not yet been evaluated. We consider our work input to conducting research in this topic, given deworming in national campaigns is not implemented in the country [18].

It is noteworthy that the coastal areas (provinces I Territorios Insulares Oceánicos del Caribe and IV Sierra Nevada de Santa Marta) have had the highest prevalence of STH infections in two of the national surveys (NMS 1966: Atlantic and Pacific Region and NSIP 2012). In the latter survey, province VIII, *La Amazonía*, was also found to have high prevalence. This could be explained by the fact that in addition to the warm, humid climate with average temperatures of 86°F and sandy soils that are advantageous to the development of eggs, these areas, compared to the rest of the country, have the lowest coverage by piped sewage systems and potable water [19].

This study demonstrated that almost all the hookworm infections, half of the *T*. *Trichiura* infections, and a third of the *A*. *lumbricoides* infections were light. Overall, almost 90% of the infections were light and moderate. This finding might be the result of the improving living conditions in the Nor-Andina region, where the highest proportion of the country’s population lives. At least one fifth of the study population performed individual deworming practices, but according our sensitivity analysis, this finding did not affect that estimate. Recent practices of health promotion in some municipalities included large-scale deworming in schools, possibly affecting the STH prevalence. This explanation is plausible since deworming is known to decrease the spreading and reduce the prevalence of STH, and, more importantly, is connected to a reduction in the intensity of infections [20,21].

The most recent national study assessing the prevalence of STH infections in the Latin-American subcontinent was conducted in Ecuador, where they found a similar STH prevalence (27,9%). The Amazonía region had the highest prevalence and presents the most similar conditions to the biogeographical regions of Colombia [22]. Additionally, a study in El Salvador was conducted in 2012 through a survey analyzing the STH prevalence by ecological zones. An overall STH prevalence of 7.6% was found, which is four times lower than that in Colombia (29.4%). Nonetheless, the prevalence by the type of STH behaved similarly in terms of dominance by species [23].

The prevalence of STH in Colombia was found to be higher in rural populations in provinces with different biogeographic, sanitary and socioeconomic conditions. This distribution is most probably linked to the fact that the highest proportion of the population with unmet basic needs (UBN) are in the areas of the country where the rates of poverty (or extreme poverty) are highest. Nearly a quarter of the population in provinces I, *Territorios Insulares Oceánicos del Caribe*, and IV, *Sierra Nevada de Santa Marta*, dispose their excreta in the open field [8,19].

We found a relative high proportion of anemia (14,2%) in contrast with very low level of undernutrition (2.1%). This could be due to nutrition factors, with high consumption of carbohydrates and low level of protein and micronutrients. The highest prevalence of anemia appeared in province I, *Territorios Insulares Oceánicos del Caribe* (45.1%) which is an oceanic island territory situated 750 km from the nearest Colombian port (Cartagena). The climate characteristics and the distance of this province from the mainland limits the population’s production, availability, and access to food, which may contribute to the highest presence of anemia in contrast to their lower STH prevalence [24].

National guidelines recommend targeting vulnerable populations using poverty criteria, concentrating the prevention and control interventions twice every year. Deworming rounds should cover at least 75% of the eligible population with each round being performed within less than twenty days. The rounds should be sustained for 6 consecutive years to interrupt the transmission of STH and achieve a greater impact on the prevalence [20].

The associations between STH and environmental factors demonstrate that prevention and control of STH are achieved not only by using preventive anthelmintic chemotherapy, but also by the proper management of living conditions that may influence the dynamics of transmission. A coordinated STH control program should establish a sentinel surveillance system of the prevalence behavior over time. It should be further accompanied by health promotion actions (hygiene habits, use of footwear, etc.) and intersectoral work aiming to improve basic sanitation conditions such as expanding piped drinking water and, sewage systems coverage, or implementing alternative solutions. In Colombia, the Ministry of Health and Social Protection, supported by the Pan American Health Organization (PAHO), has developed the ''Plan for the Prevention, Control, and Elimination of Neglected Infectious Diseases Prioritized in Colombia from 2013 to 2017'' including STH control [17,21–25].

In conclusion, the present study shows that Colombia has a moderate risk of STH infections in school-aged populations, with considerable variations in prevalence among the biogeographic provinces. Like any public health problems, this should be handled with a comprehensive approach, that includes interventions focused on reducing both the acute morbidity and long-term complications that are attributed to STH infections. As such, these interventions should promote national strategies for the control of STHs, with intersectoral action to improve the living conditions associated with this type of infection.

Pre-school and school-aged children represent a priority group in the prevention of STH infections because they are at a stage of rapid physical growth and learning. In Colombia, at least 30% of school children are affected by STH infections, and this proportion is higher than 50% in some provinces. Since STH infection has been associated with delay in weight-height growth, anemia, and poor school performance, this could perpetuate the cycle in the country, especially high-risk areas, to remain in a vicious circle of poverty and infection-reinfection [5,25].

The study encountered several limitations. Dividing the country into biogeographic provinces was the strategy used to classify the homogeneous eco-epidemiological areas by conditions that may affect STH infection prevalence. However, province V, *Chocó–Magdalena*, consists of several departments in which the living conditions vary widely. We presented prevalence by provinces, but not by departments. For that reason, differences among the conditions in each province could be masked.

The final sample size allows making inferences of the STH prevalence at a national and biogeographic province level, with a narrow confidence interval. Estimates in provinces I, *Territorios Insulares Oceánicos del Caribe*, and IV, *Sierra Nevada de Santa Marta*, were obtained with less precision, but given the frequency of the event, the confidence interval was retained without affecting the validity of inference. Since fieldwork was done in different periods of the year, the variation in the STH prevalence by biogeographic province could have been affected, to some extent, by the differences in the prevailing climate conditions. However, this could not produce a big variation in climate because of the tropical conditions in Colombia. Additionally, the data were collected during similar seasons over two years in November, February and September to November, times when variation would be limited.

Since we obtained only one stool sample per child, the STH prevalence could be underestimated, and, as a result, we used a positive result for any performed technique in the STH prevalence estimation to increase sensitivity. We also applied a formula to estimate prevalence even though the simple calculation was close, we kept adjusted than crude results.

Our work supports the design of future research from a municipal perspective, where it is necessary to determine the socioeconomic and environmental determinants that influence the persistence of STH infection in Colombian schoolchildren.

## Supporting information

S1 File STROBE checklist. (DOCX)

S2 File Proportion of missing data. (DOCX)

S3 File Prevalence of intestinal helminths. (DOCX)

S1 Fig Biogeographic provinces in Colombia and municipalities visited. (TIF)

S2 Fig STH infection Prevalence by Biogeographic province. (TIF)

S3 Fig Prevalence of STH. (TIF)

S1 Table School participants by province. Colombia 2012–2013.
(DOCX)

S2 Table Soil Transmitted Helminthic Infection, Anemia and Nutritional Status at school-aged participants by province. Colombia 2012–2013.
(DOCX)
