# Supplementary material for: Prevalence of soil transmitted helminths in school-aged children, Colombia, 2012-2013
Source: PLoS Negl Trop Dis. 2020 Jul 17;14(7):e0007613. doi: 10.1371/journal.pntd.0007613 (PMC7390406; doi:10.1371/journal.pntd.0007613)
Supplement: S1 File — (DOCX) [file pntd.0007613.s001.docx]

STROBE Statement—Checklist of items that should be included in reports of ***cross-sectional studies***.

|  | Item No | Recommendation | Page-Lines |
| --- | --- | --- | --- |
| **Title and abstract** | 1 | (*a*) Indicate the study’s design with a commonly used term in the title or the abstract | Pag 1, Lines 1 – 2 |
|  |  | (*b*) Provide in the abstract an informative and balanced summary of what was done and what was found | Pag 1 – 2, Lines 25 – 55 |
| Introduction | | |  |
| Background/rationale | 2 | Explain the scientific background and rationale for the investigation being reported | Pag 2, Lines 76 – 94 |
| Objectives | 3 | State specific objectives, including any prespecified hypotheses | Pag 3 , Lines 95 – 98 |
| Methods | | |  |
| Study design | 4 | Present key elements of study design early in the paper | Pag 3, Lines 102 -104 |
| Setting | 5 | Describe the setting, locations, and relevant dates, including periods of recruitment, exposure, follow-up, and data collection | Pag 3 – 4, Lines 106 – 157 |
| Participants | 6 | (*a*) Give the eligibility criteria, and the sources and methods of selection of participants | Pag 4, Lines 159 – 163 |
| Variables | 7 | Clearly define all outcomes, exposures, predictors, potential confounders, and effect modifiers. Give diagnostic criteria, if applicable | Pag 5 – 6, Lines 207 – 231  Pag 7 - 8, Lines 307 – 316 |
| Data sources/ measurement | 8* | For each variable of interest, give sources of data and details of methods of assessment (measurement). Describe comparability of assessment methods if there is more than one group | Pag 5 – 6, Lines 207 – 231 |
| Bias | 9 | Describe any efforts to address potential sources of bias | Pag 6, Lines 243 - 245  Pag 7 - 8, Lines 304 – 321 |
| Study size | 10 | Explain how the study size was arrived at | Pag 4 – 5, Lines 165 – 205 |
| Quantitative variables | 11 | Explain how quantitative variables were handled in the analyses. If applicable, describe which groupings were chosen and why | Pag 5 - 6, Lines 216 - 223 |
| Statistical methods | 12 | (*a*) Describe all statistical methods, including those used to control for confounding | Pag 7 – 8, Lines 285 – 331 |
|  |  | (*b*) Describe any methods used to examine subgroups and interactions | Pag 7 – 8, Lines 285 – 331 |
|  |  | (*c*) Explain how missing data were addressed | S1 Table |
|  |  | (*d*) If applicable, describe analytical methods taking account of sampling strategy | Pag 7 – 8, Lines 285 – 331 |
|  |  | (*e*) Describe any sensitivity analyses | Pag 8, Lines 318 - 321 |
| Results | | |  |
| Participants | 13* | (a) Report numbers of individuals at each stage of study—eg numbers potentially eligible, examined for eligibility, confirmed eligible, included in the study, completing follow-up, and analysed | See Table 1 |
|  |  | (b) Give reasons for non-participation at each stage | Pag 4, Lines 159 - 163 |
|  |  | (c) Consider use of a flow diagram | See Table 1 |
| Descriptive data | 14* | (a) Give characteristics of study participants (eg demographic, clinical, social) and information on exposures and potential confounders | Pag 8 – 10, Lines 337 – 393 and Table 2 |
|  |  | (b) Indicate number of participants with missing data for each variable of interest | S1 Table |
| Outcome data | 15* | Report numbers of outcome events or summary measures | Fig 2,3, 4a-c y 5a-c and Pag 11 Lines 396 – 419 |
| Main results | 16 | (*a*) Give unadjusted estimates and, if applicable, confounder-adjusted estimates and their precision (eg, 95% confidence interval). Make clear which confounders were adjusted for and why they were included | Table 3. Lines 422 – 426  S2 Table |
|  |  | (*b*) Report category boundaries when continuous variables were categorized | Pag 7 - 8, Lines 307 - 316 |
|  |  | (*c*) If relevant, consider translating estimates of relative risk into absolute risk for a meaningful time period | NA |
| Other analyses | 17 | Report other analyses done—eg analyses of subgroups and interactions, and sensitivity analyses | Pag 8, Lines 318 – 321 |
| Discussion | | |  |
| Key results | 18 | Summarise key results with reference to study objectives | Pag 13, Lines 472 – 488 |
| Limitations | 19 | Discuss limitations of the study, taking into account sources of potential bias or imprecision. Discuss both direction and magnitude of any potential bias | Pag 16, Lines 605 – 619 |
| Interpretation | 20 | Give a cautious overall interpretation of results considering objectives, limitations, multiplicity of analyses, results from similar studies, and other relevant evidence | Pag 13 - 16, Lines 489 – 623 |
| Generalisability | 21 | Discuss the generalisability (external validity) of the study results | Pag 16, Lines 612 – 616 |
| Other information | | |  |
| Funding | 22 | Give the source of funding and the role of the funders for the present study and, if applicable, for the original study on which the present article is based |  |
