# Supplementary material for: Prevalence of soil transmitted helminths in school-aged children, Colombia, 2012-2013
Source: PLoS Negl Trop Dis. 2020 Jul 17;14(7):e0007613. doi: 10.1371/journal.pntd.0007613 (PMC7390406; doi:10.1371/journal.pntd.0007613)
Supplement: S2 File — (DOCX) [file pntd.0007613.s002.docx]

**S1 Table. Proportion of missing data.**

| **Variables** | **Frequency** | **%** | **Missing** | **%** |
| --- | --- | --- | --- | --- |
| Socioeconomic status | 7732 | 88.7 | 128 | 1.6 |
| Affiliation with social security in health | 7793 | 99.1 | 67 | 0.9 |
| Hemoglobin value | 7793 | 99.2 | 67 | 0.9 |
| Insects and rodents near the house | 7809 | 99.4 | 51 | 0.6 |
| Playing with dirt | 7823 | 99.5 | 37 | 0.5 |
