# Supplementary material for: Prevalence of soil transmitted helminths in school-aged children, Colombia, 2012-2013
Source: PLoS Negl Trop Dis. 2020 Jul 17;14(7):e0007613. doi: 10.1371/journal.pntd.0007613 (PMC7390406; doi:10.1371/journal.pntd.0007613)
Supplement: S3 File — (DOCX) [file pntd.0007613.s003.docx]

**S2 Table. Prevalence of intestinal helminths.**

| **Helminth** | **Estimator** | **National value** | **Biogeographic Province** | | | | | | | |
| --- | --- | --- | --- | --- | --- | --- | --- | --- | --- | --- |
|  |  |  | **I.** | **III.** | **IV.** | **V.** | **VI.** | **VII.** | **VIII.** | **IX.** |
| *A.lumbricoides* | Prevalence | **11.3** | 2.0 | 20.1 | 42.9 | 16.7 | 0.2 | 0.6 | 58.0 | 3.2 |
|  | CI | 7.1 - 17.4 | 0.5 - 7.8 | 14.6 - 27.1 | 37.5 - 48.5 | 7.8 - 32.2 | 0 - 2.9 | 0.5 - 0.8 | 51.3 - 64.3 | 1.4 - 7.1 |
|  | Error | 2.5 | 1.4 | 3.1 | 2.8 | 6 | 0.3 | 0.1 | 3.3 | 1.3 |
| *T. trichiura* | Prevalence | **18.4** | 9.8 | 42.7 | 61.0 | 30.0 | 14.5 | 4.8 | 50.0 | 4.3 |
|  | CI | 11.8 - 27.7 | 5.5 - 16.9 | 31.5 - 54.6 | 55.9 - 65.8 | 15.2 - 50.5 | 10.2 - 20.3 | 4.1 - 5.6 | 45.9 - 54.2 | 1.5 - 11.6 |
|  | Error | 4 | 2.8 | 5.9 | 2.5 | 9.1 | 2.5 | 0.4 | 2.1 | 2.2 |
| *Hookworms* | Prevalence | **6.4** | 0.0 | 13.2 | 3.4 | 8.9 | 9.8 | 10.8 | 35.7 | 1.0 |
|  | CI | 3.7 - 10.8 | 0 – 0 | 4.9 - 30.8 | 2.2 - 5.1 | 3.5 - 20.7 | 3 - 27.5 | 10.1 - 11.5 | 32.4 – 39 | 0.2 - 4.9 |
|  | Error | 1.7 | 0 | 6.1 | 0.7 | 4 | 5.5 | 0.4 | 1.6 | 0.8 |
| *S. stercoralis* | Prevalence | **0.7** | 0.0 | 0.6 | 1.4 | 0.9 | 0.0 | 0.8 | 4.4 | 0.3 |
|  | CI | 0.4 - 1.1 | 0 – 0 | 0.3 - 1.4 | 0.8 - 2.7 | 0.4 - 1.9 | 0 - 0.3 | 0.5 - 1.4 | 3.8 - 5.1 | 0.1 - 1 |
|  | Error | 0.2 | 0 | 0.2 | 0.5 | 0.4 | 0 | 0.2 | 0.3 | 0.2 |
| *E. vermicularis* | Prevalence | **1.0** | 0.0 | 0.3 | 0.0 | 1.0 | 4.6 | 6.4 | 0.3 | 0.8 |
|  | CI | 0.6 - 1.8 | 0 – 0 | 0.1 - 1.2 | 0 - 0 | 0.3 - 3.3 | 2.3 - 8.9 | 6 - 6.9 | 0.2 - 0.5 | 0.4 - 1.8 |
|  | Error | 0.3 | 0 | 0.2 | 0 | 0.6 | 1.6 | 0.2 | 0.1 | 0.3 |
| *H. nana* | Prevalence | **0.9** | 0.0 | 4.2 | 11.0 | 0.5 | 0.4 | 0.0 | 3.6 | 0.4 |
|  | CI | 0.6 - 1.4 | 0 – 0 | 2.3 - 7.6 | 9.8 - 12.4 | 0.2 - 1.4 | 0.1 - 2.5 | 0 - 0 | 2.4 - 5.2 | 0.2 - 1 |
|  | Error | 0.2 | 0 | 1.3 | 0.6 | 0.3 | 0.4 | 0 | 0.7 | 0.2 |
