# Supplementary figures and images for: Prevalence of soil transmitted helminths in school-aged children, Colombia, 2012-2013
Source: PLoS Negl Trop Dis. 2020 Jul 17;14(7):e0007613. doi: 10.1371/journal.pntd.0007613 (PMC7390406; doi:10.1371/journal.pntd.0007613)

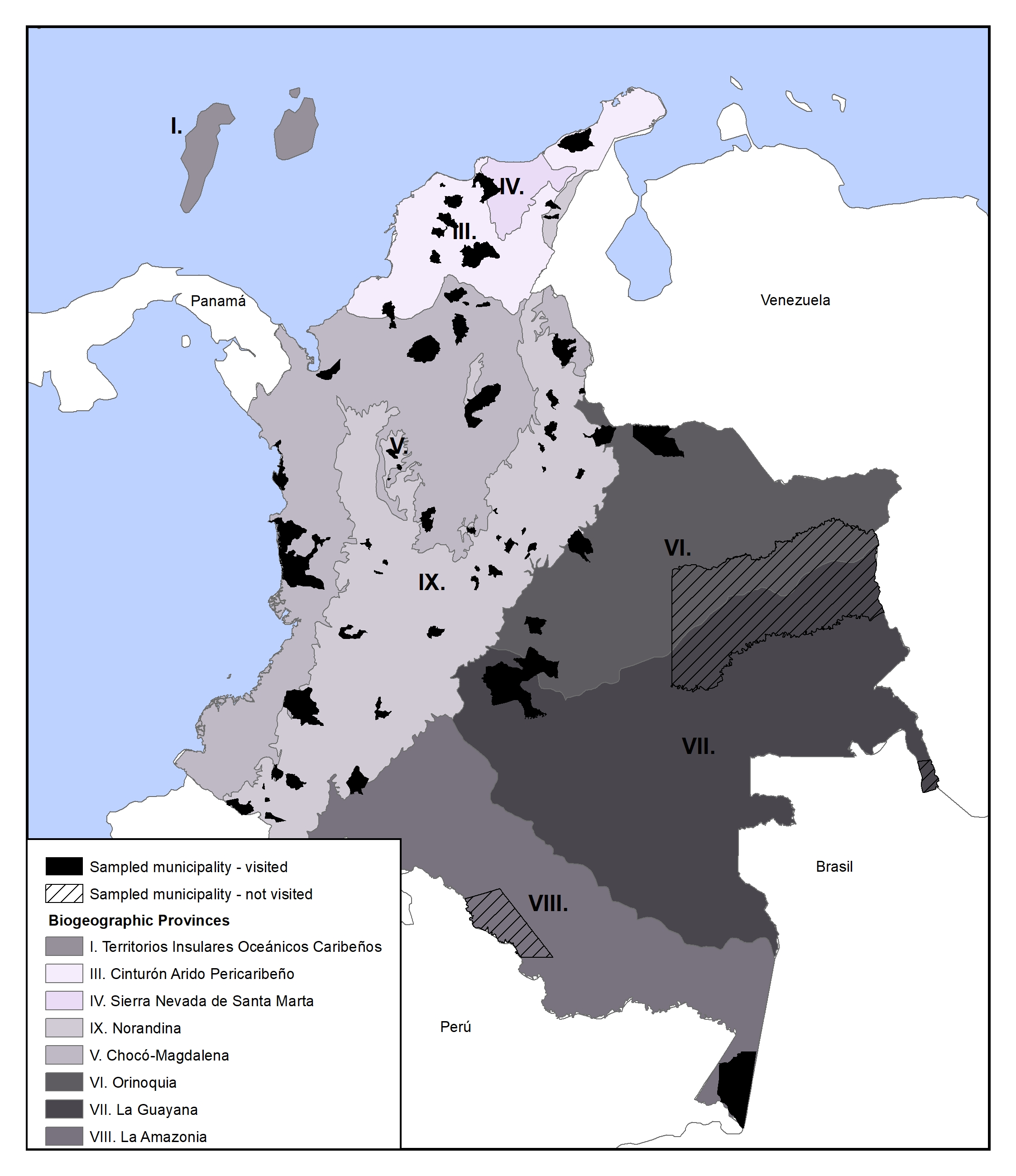

Supplement: S1 Fig — (TIF) [file pntd.0007613.s004.tif]

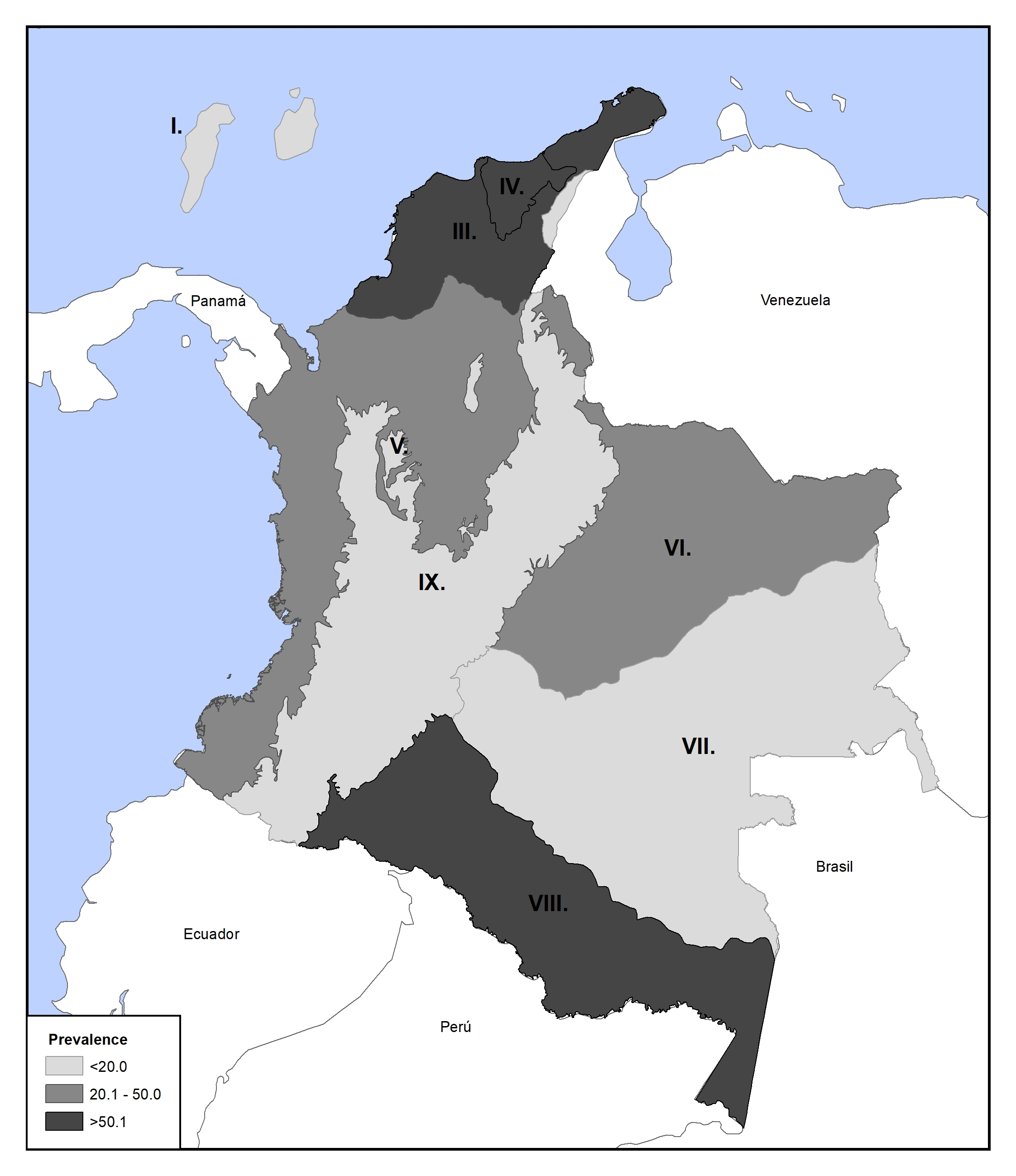

Supplement: S2 Fig — (TIF) [file pntd.0007613.s005.tif]

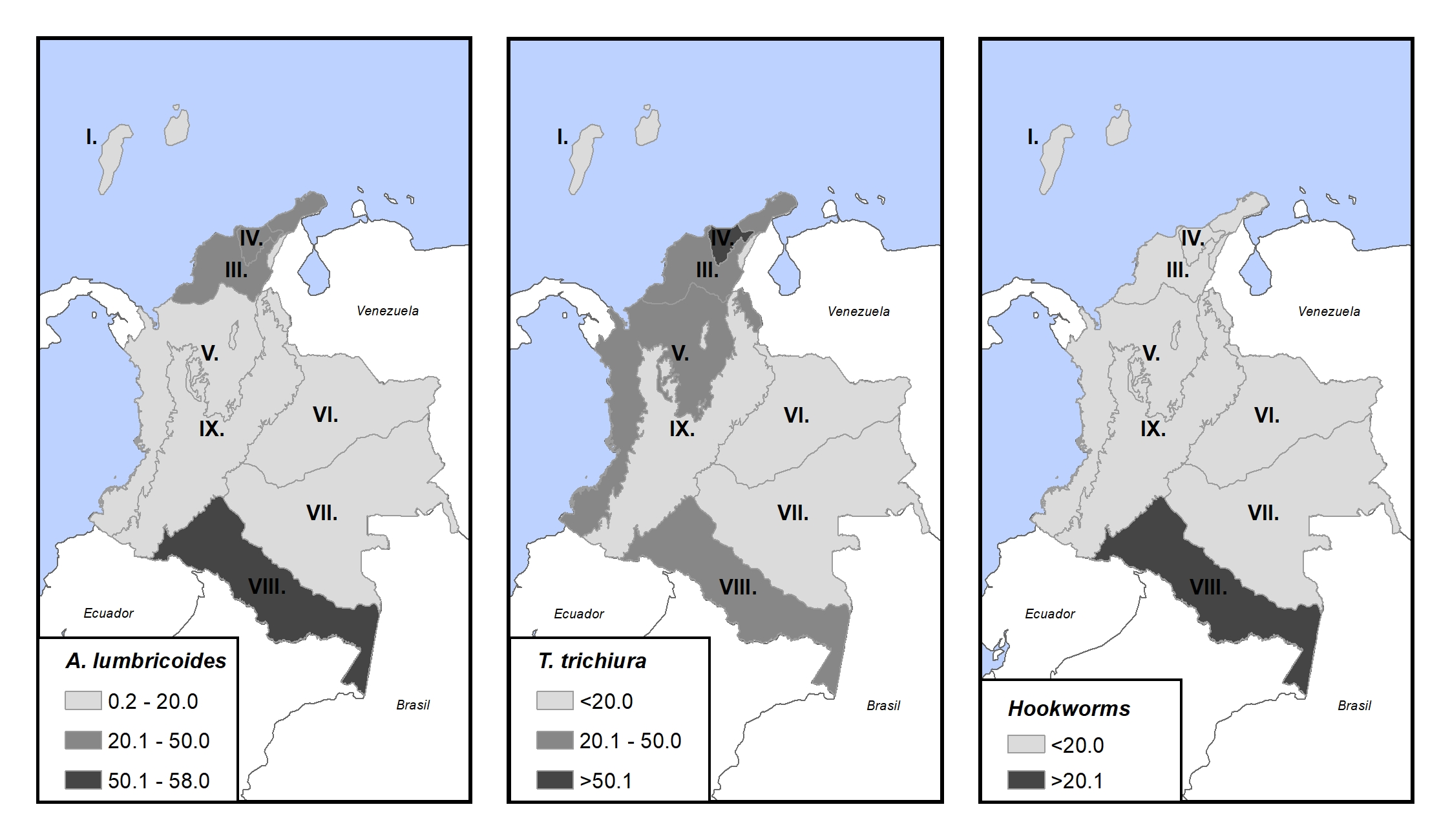

Supplement: S3 Fig — (TIF) [file pntd.0007613.s006.tif]
