# Supplementary material for: Prevalence of soil transmitted helminths in school-aged children, Colombia, 2012-2013
Source: PLoS Negl Trop Dis. 2020 Jul 17;14(7):e0007613. doi: 10.1371/journal.pntd.0007613 (PMC7390406; doi:10.1371/journal.pntd.0007613)
Supplement: S1 Table — Colombia 2012–2013. (DOCX) [file pntd.0007613.s007.docx]

S1 Table . School participants by province. Colombia 2012-2013.

| **Province** | **Number of schools (visited/total schools on selected municipalities)** | **School children, 7-10 years old*** | **n   (expected)** | **n (sampled)** |
| --- | --- | --- | --- | --- |
| **I. Territorios Insulares Oceánicos del Caribe** | 2 / 3 | 4567 | 320 | 36 |
| **III. Cinturón Árido Pericaribeño** | 21 / 96 | 391990 | 341 | 994 |
| **IV. Sierra Nevada de Santa Marta** | 3 / 21 | 62219 | 340 | 75 |
| **V. Chocó-Magdalena** | 41 / 527 | 515693 | 1642 | 1570 |
| **VI. La Orinoquía** | 9 / 122 | 107885 | 1531 | 322 |
| **VII. La Guayana** | 3 / 11 | 29048 | 911 | 155 |
| **VIII. La Amazonía** | 3 / 30 | 44129 | 390 | 104 |
| **IX. Nor-Andina** | 91 / 736 | 1084635 | 653 | 2789 |
| **Total** | **173 / 1546** | **2240165** | **6128** | **6045** |

* Estimation based on population projections for DANE, Colombia. Population projections. School-aged children, 2012
