# Supplementary material for: Prevalence of soil transmitted helminths in school-aged children, Colombia, 2012-2013
Source: PLoS Negl Trop Dis. 2020 Jul 17;14(7):e0007613. doi: 10.1371/journal.pntd.0007613 (PMC7390406; doi:10.1371/journal.pntd.0007613)
Supplement: S2 Table — Colombia 2012–2013. (DOCX) [file pntd.0007613.s008.docx]

S2 Table . Soil Transmitted Helminthic Infection, Anemia and Nutritional Status at school-aged participants by province. Colombia 2012-2013.

|  | **I. Territorios Insulares Oceánicos del Caribe** | **III. Cinturón Árido Pericaribeño** | **IV. Sierra Nevada de Santa Marta** | **V. Chocó-Magdalena** | **VI. La Orinoquía** | **VII. La Guayana** | **VIII. La Amazonía** | **IX. Nor-Andina** | **National** |
| --- | --- | --- | --- | --- | --- | --- | --- | --- | --- |
|  |  |  |  |  |  |  |  |  |  |
| **PREVALENCE STH** | **%  (95% CI)** | **%  (95% CI)** | **%  (95% CI)** | **%  (95% CI)** | **%  (95% CI)** | **%  (95% CI)** | **%  (95% CI)** | **%  (95% CI)** | **%  (95% CI)** |
| ***Ascaris lumbricoides*** | 2  (0.5-7.8) | 20.1  (14.6-27.1) | 42.9  (37.5-48.5) | 16.7  (7.8-32.2) | 0.2  (0-2.9) | 0.6  (0.5-0.8) | 58  (51.3-64.3) | 3.2  (1.4-7.1) | **11.3   (7.1 - 17.4)** |
| ***Trichuris trichiura*** | 9.8 (5.5-16.9) | 42.7 (31.5-54.6) | 61 (55.9-65.8) | 30 (15.2-50.5) | 14.5 (10.2-20.3) | 4.8 (4.1-5.6) | 50 (45.9-54.2) | 4.3 (1.5-11.6) | **18.4 (11.8 - 27.7)** |
| **Hookworms** | 0.0 | 13.2 (4.9-30.8) | 3.4 (2.2-5.1) | 8.9 (3.5-20.7) | 9.8 (3-27.5) | 10.8 (10.1-11.5) | 35.7 (32.4-39) | 1 (0.2-4.9) | **6.4 (3.7 - 10.8)** |
| **All STH** | 10.9 | 56.8 | 74 | 44.2 | 21.7 | 14.7 | 81.6 | 7.8 | **29.6** |
| **INTENSITY STH** |  | | | | | | | | |
| ***Ascaris lumbricoides*** |  |  |  |  |  |  |  |  |  |
| Light | 100 | 54 (47.6-60.5) | 16.4 (9.2-27.7) | 37.8 (22.5-56) | 0.0 | 100 | 26 (21.9-30.6) | 59.5 (40-76.4) | **39.9 (30.7 - 49.9)** |
| Moderate | 0.0 | 38.4 (30.9-46.5) | 53.7  (48.4-58.9) | 43 (32.1-54.5) | 100 | 0.0 | 39 (37.8-40.2) | 38  (22.9-55.8) | **41.3 (35.0 - 47.8)** |
| Heavy | 0.0 | 7.5 (4.9-11.3) | 29.8 (25.1-35.1) | 19.2 (11.6-30) | 0.0 | 0.0 | 35 (31.4-38.8) | 2.5 (0.6-9.2) | **18.8 (14.1 - 24.6)** |
| ***Trichiuris trichiura*** |  |  |  |  |  |  |  |  |  |
| Light | 60 (26-86.5) | 64.2 (58.8-69.2) | 61.4 (56.7-65.9) | 52.8  (40.3-64.9) | 80.3 (49.3-94.5) | 69.6 (62.9-75.5) | 56.7 (53.8-59.5) | 76.9 (65-85.6) | **59 (50.5 - 66.9)** |
| Moderate | 40 (13.5-74) | 34 (28.9-39.5) | 37 (32.8-41.4) | 41.1 (31.4-51.5) | 19.7 (5.5- 50.7) | 30.4 (24.5-37.1) | 40.4 (38-42.9) | 22.9 (14.2-34.9) | **36.9 (30.5 - 43.8)** |
| Heavy | 0.0 | 1.9 (0.7-4.6) | 1.6  (0.7-3.7) | 6.2  (3.7-10.1) | 0.0 | 0.0 | 2.9 (2.4-3.6) | 0.2 (0-1.8) | **4.1 (2.5 - 6.60)** |
| **Hookworms** |  | | | | | | | | |
| Light | 0.0 | 81.4  (68.7-89.8) | 71.4 (50.7-85.8) | 83.7 (73.1-90.6) | 100 | 100 | 85.7 (84.1-87.1) | 99.6 (94.8-100) | **86.4 (79.8 - 91.1)** |
| Moderate | 0.0 | 9.7 (4.4-20.1) | 28.6 (14.2-49.3) | 9.4 (5.7-15.3) | 0.0 | 0.0 | 8.2 (6.8-9.9) | 0.4 ( 0-5.2) | **7.7 (5.2 - 11.5)** |
| Heavy | 0.0 | 8.9 (5.7-13.5) | 0.0 | 6.9 (3.5-13.1) | 0.0 | 0.0 | 6.1 (5.5-6.9) | 0.0 | **5.9 (3.7 - 9.3)** |
| **PREVALENCE** |  |  |  |  |  |  |  |  |  |
| **Anemia** | 45.1 (35.3 - 55.4) | 30.6 (24.4 - 37.7) | 18.1 (15.3 - 21.3) | 24.6 (15.2 - 37.3) | 30.5 (27 - 34.3) | 25.1 (24.4 - 25.9) | 29.6 (28 - 31.4) | 3.5 (2.1 - 5.8) | **14.2 (9.4 - 20.9)** |
| **Nutritional Status** | |  |  |  |  |  |  |  |  |
| Obesity | 16.7  (6.3-32.8) | 1.4  (0.6-2.2) | 0.0 | 5.4  (4.2-6.5) | 9.0  (5.8-12.3) | 4.5  (1.8-9.0) | 6.7  (2.7-13.3) | 7.1  (6.2-8.1) | **5.8**  **(5.1-6.3)** |
| Overweight | 8.3  (1.7-22.4) | 7.4  (5.8-9.1) | 4.0  (0.8-11.2) | 12.6  (10.8-14.2) | 18.9  (14.2-22.9) | 14.1  (9.1-20.6) | 14.4  (8.3-22.6) | 16.2  (14.4-17.5) | **13.7**  **(12.7-14-4)** |
| Normal | 75.0  (57.7-87.8) | 87.0  (86.2-90.3) | 92.0  (83.3-87.0) | 78.8  (77.1-81.2) | 71.1  (65.8-76.0) | 81.2  (74.2-87.0) | 77.8  (68.6-85.4) | 75.8  (74.2-77.4) | **78.9**  **(77.8-79.9)** |
| Thinness | 0.0 | 2.7  (1.7-3.8) | 2.7  (0.3-9.3) | 1.2  (0.7-1.8) | 1.2  (0.3-3.1) | 0.0 | 1.0  (0.02-5.2) | 0.8  (0.4-1.1) | **1.2**  **(1-1.5)** |
| Severe thinness | 0.0 | 0.2  (0.06-0.9) | 1.3  (0.00-7.2) | 0.0 | 0.0 | 0.0 | 0.0 | 0.0 | **0.6**  **(0.4-0.8)** |
